# Desmoplastic Small Round Blue Cell Tumor: A Review of Treatment and Potential Therapeutic Genomic Alterations

**DOI:** 10.1155/2017/1278268

**Published:** 2017-11-01

**Authors:** Ajaz Bulbul, Bridget Noel Fahy, Joanne Xiu, Sadaf Rashad, Asrar Mustafa, Hatim Husain, Andrea Hayes-Jordan

**Affiliations:** ^1^Department of Hematology/Oncology, Kymera Independent Physicians, Carlsbad, NM, USA; ^2^Division of Internal Medicine, Department of Hematology/Oncology, Texas Tech University Health Sciences Center School of Medicine, Lubbock, TX, USA; ^3^Department of Surgery, University of New Mexico, Albuquerque, NM, USA; ^4^Caris Life Sciences, Phoenix, AZ, USA; ^5^All Saints University School of Medicine, Roseau, Dominica; ^6^Acharya Shri Chander College of Medical Sciences and Hospital, Jammu, India; ^7^Moores Cancer Center, University of California, San Diego, La Jolla, CA, USA; ^8^Department of Pediatric Surgical Oncology, University of Texas MD Anderson Cancer Center, Houston, TX, USA

## Abstract

Desmoplastic small round blue cell tumors (DSRCTs) originate from a cell with multilineage potential. A molecular hallmark of DSRCT is the EWS-WT1 reciprocal translocation. Ewing sarcoma and DSRCT are treated similarly due to similar oncogene activation pathways, and DSRCT has been represented in very limited numbers in sarcoma studies. Despite aggressive therapy, median survival ranges from 17 to 25 months, and 5-year survival rates remain around 15%, with higher survival reported among those undergoing removal of at least 90% of tumor in the absence of extraperitoneal metastasis. Almost 100% of these tumors contain t(11;22) (p13;q12) translocation, and it is likely that EWS-WT1 functions as a transcription factor possibly through WT1 targets. While there is no standard protocol for this aggressive disease, treatment usually includes the neoadjuvant HD P6 regimen (high-dose cyclophosphamide, doxorubicin, and vincristine (HD-CAV) alternating with ifosfamide and etoposide (IE) chemotherapy combined with aggressively attempted R0 resection). We aimed to review the molecular characteristics of DSRCTs to explore therapeutic opportunities for this extremely rare and aggressive cancer type.

## 1. Background

Desmoplastic small round blue cell tumors (DSRCTs) are a highly aggressive and rare mesenchymal tumor, of which approximately 200–450 cases have been described so far [1, 2]. DSRCT was first reported as a separate identity in 1989 by Gerald and Rosai [3] who proposed that DSRCT arose during development from a progenitor cell with potential for multiphenotypic differentiation [4].

## 2. Clinical Presentation

Young men comprise the vast majority of cases with a mean age at diagnosis of 22 years [2, 5, 6]. Clinically, DSRCT has been shown to have a predilection for developing in the abdominal and pelvic cavity with extra-abdominal involvement being much less common [5].

In the majority of cases, patients with DSRCT present with advanced disease. Most patients remain asymptomatic for extended periods of time, and diagnosis is made when tumor burden is significant [2]. The most common symptoms are abdominal pain and weight loss [5]. Constipation due to mass effect caused by the tumor and bowel obstruction have also been reported [2]. Due to a significant burden of peritoneal disease, some patients will present with an abdominal mass alone, but the most common presentation is abdominal distension from ascites. Liver metastases are seen both at the time of diagnosis and with relapse. Other distant sites include lymph nodes, lungs, and bones [2, 7, 8]. Omental and hepatic metastases can also be seen.

## 3. Histopathology

Histologically, the tumors consist of solid sheets, large nests, small clumps, or cords of cohesive, small, round, ovoid, or spindled cells lying in a hypocellular, desmoplastic, collagenous stroma [5]. Immunohistochemical staining demonstrates the divergent differentiation of the neoplastic cells. Neoplastic cells typically express epithelial (keratin and epithelial membrane antigen (EMA)), mesenchymal (vimentin), neural (CD56 and neuron-specific enolase (NSE)), and muscle (desmin) markers [4, 9]. The molecular hallmark of DSRCT is the EWS-WT1 fusion protein. The t(11;22) (p13;q12) translocation is present in virtually all cases [10–12]. The WT1 protein is a transcriptional activator of genes involved in renal and gonadal differentiation; it regulates the mesenchymal to epithelial transition that occurs in renal development. Most of these tumors contain t(11;22) (p13;q12) translocation, and it is possible that EWS-WT1 functions as a transcription factor, possibly through WT1 targets [13, 14].

## 4. Diagnostic Studies

CT scan with oral and intravenous contrast is the imaging modality of choice when evaluating patients with known or suspected DSRCT. Characteristic findings in DSRCT include soft tissue masses which are often bulky (mean, 6 cm; range, 1–28 cm), lobulated, and heterogeneous with hypodense areas; these findings are present in up to three-quarters of patients [15], and a significant desmoplastic reaction differentiates DSRCTs from other small round cell tumors [16]. Adenopathies are present in approximately half of patients at the time of diagnosis (intraperitoneal, retroperitoneal, and pelvic) [15, 17]. In a radiological review of 13 cases of abdominal DSRCT, the most common finding was the presence of several lobulated peritoneal soft tissue masses (mean number of masses/patient = 4). The main sites of peritoneal involvement were the pelvis, omentum, retroperitoneum, and small bowel mesentery. In six cases, moderate ascites was seen. Five of 13 patients had liver metastases, with an average of four lesions per case. Associated thoracic metastases were found in three patients [18].

MRI findings include heterogeneous T1 low signal and heterogeneous T2 high signal. After gadolinium administration, there is heterogeneous contrast enhancement. Subtle hypointense foci are sometimes seen on T2-weighted images representing desmoplastic reaction. Hyperintense T1 signal and fluid/fluid levels may suggest recent hemorrhage in a tumor [19].

FDG-PET scanning has been shown to impart important additional information and has a relevant impact on treatment planning when used in concert with CT scan [20, 21]. FDG-PET is sometimes obtained at diagnosis and during surveillance. FDG-PET/CT has been found to be superior to CT in detection of lymph node involvement (sensitivity, 95% versus 25%, resp.), bone lesions (sensitivity, 90% versus 57%, resp.), and renal lesions [20]. In a study of 65 patients, FDG uptake was seen in all primary intra-abdominal and pelvic tumors [22] and accurately detected 97% of all DSRCT lesions with sensitivity, specificity, positive, and negative predictive values of 96%, 99%, 98%, and 97%, respectively. However, CT is more reliable than FDG-PET in depicting lung metastases [20].

Core biopsy specimens are preferred to acquire sufficient sample. Fine-needle aspiration specimens, although commonly employed, are not adequate during the workup of DSRCT due to issues with low cellularity of the sample, necrosis, and predominantly a desmoplastic reaction. Fine-needle aspiration is challenging and requires pathological expertise in the utilization of ancillary techniques such as immunocytochemistry and flow cytometric immunophenotyping. The absence of the characteristic desmoplastic stroma in DSRCT and its cytologic features make cytologic interpretation difficult [23]. Characteristic cytologic features are seen in the right clinical context of small round blue cells with cytoplasmic densities and purple-stained connective stroma [24] and should raise suspicion of DSRCT that should be confirmed by its unique cytogenetic abnormality. The cells include granular chromatin and smooth to irregular nuclear membranes and show nuclear molding, cytoplasmic vacuoles, pseudorosettes, and metachromatic stroma [25] compared to other potential diagnoses like Ewing sarcoma. RT-PCR for EWS-WT1 transcript detection is a way of increasing diagnostic accuracy [26]. However, using a combination of both techniques, 86.4% of DSRCT can be typed accurately [26, 27].

## 5. Staging

The UICC staging of sarcoma is inadequate for DSRCT as it classifies nearly all patients as metastatic [2]. Several staging methods have been proposed for DSRCT, and there is currently no validated staging system. Due to the extensive nature of the peritoneal disease frequently present, the Peritoneal Cancer Index is often used. In this system, the abdominal cavity is divided into 13 regions, and each region is assigned a lesion size score ranging from 0 (no tumor seen) to 3 (tumor >5 cm or confluence) [28]. The MD Anderson group has suggested the inclusion of liver and extra-abdominal metastases into this staging system to adjust for the use of hyperthermic intraperitoneal chemotherapy (HIPEC) and the finding that extra-abdominal metastasis correlated with poor survival [29]. It is unclear if this strategy applies to the adult population since the median age in that study was 12 years in few who underwent HIPEC. The estimated median overall 3-year survival for patients not undergoing surgery or HIPEC was 26% compared with 71% in patients who underwent HIPEC and surgery compared with 62% who only received debulking surgery [7].

## 6. Imaging

CT scan with contrast is the imaging of choice for staging and surveillance. CT is more reliable than FDG-PET in depicting lung metastases [20]. Soft tissue masses seen are often bulky (mean, 6 cm; range, 1–28 cm), lobulated, and heterogeneous with hypodense areas up to three-quarters of patients. Adenopathy is present in about half the time of the diagnosis (intraperitoneal, retroperitoneal, and pelvic). Occasionally, moderate ascites is seen [18]. FDG-PET scanning has been shown to impart important additional information and has a relevant impact on changing treatment planning when used in concert with CT scan [20, 30]. FDG-PET can be used at diagnosis and during surveillance and has been found to be superior to CT in detection of lymph node involvement (sensitivity, 95% versus 25%, resp.) and bone metastases (sensitivity, 90% versus 57%, resp.) [20].

## 7. Molecular Findings

As in certain other tumors, the function of the Wilms tumor protein (WT1) in repressing gene transcription is lost in DSRCT [31]. There is reported loss of the zinc finger region of WT1 in EWS-WT1 which serves to convert WT1 from a repressor of transcription to a dominant transcriptional activator oncogene including some 35 target genes [14, 32]. Some of these are growth factor genes such as *PDGFα*; growth factor receptor genes such as IGF-1 receptor, EGFR, and *IL-2/15Rβ* [13, 33]; transcriptional regulators including *c-MYC*, *n-MYC*, *PAX2-2*, *ENT4*, and *WT-1*; and extracellular protein-encoding genes such as e-Syndecan, E-cadherin, and TALLA-1 which is a tetraspanin-family genes that encodes transmembrane proteins responsible for regulating cell adhesion, migration, and metastasis [34]. CCN2 (connective tissue growth factor) is highly expressed in DSRCT and may have autocrine or paracrine roles in disease progression [31]; however, the precise contribution of these molecular events and their potential as a therapeutic target remain poorly understood and applied.

Embryologically, WT1 is involved in urogenital development [35]. Normal WT1 protein is expressed in tissues which undergo mesenchymal-epithelial conversion from mesoderm [36] and may have a role in mesothelial formation in embryonic development [37]. This may explain the mesenchymal propensity of the tumor and some usage of the name “mesothelioblastoma.” Variant fusion isoforms generated due to alternative mRNA splicing have led to discovery of two protein isoforms. Some of these cases express full-length WT1 or have variant transcripts (KTS+), resulting in atypical staining patterns. The KTS+ variant has different transcriptional targets than the KTS− isoform [38].

Neither isoform of EWS-WT1 is sufficient to transform wild-type murine embryonic fibroblasts (MEFs). The oncogenic potential of both can be unmasked by p53 loss as seen by nuclear localization of p53, and copy-number amplification and gene-set enrichment analysis demonstrated augmentation of the WNT pathway [39]. In the absence of intact p53 protein, WT1 acts as a transcriptional activator [40].

Genomic analysis on LMS, UPS, and MPNST has shown novel genetic alterations; however, DSRCT has been represented in very limited numbers [41]. Limited sequencing studies have been performed on DSRCT because of the small number of cases shown. Protein biomarkers show c-kit in 19% of cases, and HER2/neu overexpression (3+) is also seen but uncommon in desmoplastic small round cell tumors [42]. EGFR gene amplification occurs at a rate of 16.9% by FISH. This was also true for LMS, MPNST, osteosarcoma, and UPS [41].

Molecular profiling on 35 DSRCTs sampled from patients having surgery for DSRCT (Caris Life Sciences, Phoenix, AZ) that were compared with Ewing sarcoma revealed low immunogenicity (<10 mutations/Mb) and low frequency of actionable mutations including PD-L1 in both tumor types. High AR expression could present as a potential therapeutic target for DSRCT while taxanes may be more effective in Ewing sarcoma compared to DSRCT based on TUBB3 expression [43]. Given the male predominance of this subset of disease, it is not surprising that, when compared to Ewing sarcoma, no significant difference was seen in protein expressions with the exception of a significantly higher overexpression of AR in DSRCT (59% versus 3%, *p*=1.7E-10) and TUBB3 (56% versus 29%, *p*=0.03) [43].

There is known to be relatively low concordance across platforms and for individual genes or proteins. cKIT overexpression by IHC in one study did not associate with *cKIT* mutations [41]. This is in contrast to GIST, where more than 80% of cases carry an activating mutation in the KIT gene and more concordance is seen [44]. There is in fact quite a low frequency of actionable mutations detected in series that looked at genomic alterations which overall included only 9 patients of DSRCT [41].

Given the interest in immunotherapy, currently, most of the interest lies with PD-1 and PD-L1 inhibitors. Previous work has shown that both PD-1 positivity and PD-L1 positivity were independent prognostic indicators of OS and EFS in sarcoma [45]. Intratumoral infiltration of PD-1-positive lymphocytes and PD-L1 expression have been seen in 65% and 58% of STS, respectively [45]. PD-1 positivity and PD-L1 expression are associated with advanced clinicopathological parameters and presence of distant metastasis, and both PD-1 positivity and PD-L1 positivity are independent prognostic indicators of overall survival (OS) [45, 46]. Over 150 sarcoma subtypes have been analyzed for PD-L1 tumor expression and the presence of PD-1+ tumor-infiltrating lymphocytes (TILs): up to 65% of sarcomas expressed PD-L1 which, along with PD-1 TIL positivity, correlates with poorer overall survival and aggressive tumor features [47]. DSRCT, however, is not very well represented in these studies.

We now know that a higher mutational rate is observed in melanoma (median of 13.2 mutations per Mb) and in NSCLC, reflecting their high responses to immunotherapy. The median of somatic mutations per Mb is 10.5 for smokers and 0.6 for nonsmokers, in which mutations are known to be secondary caused by selective pressures such as UV light and tobacco smoke exposure, respectively.

Mutational loads are lower in MSS colorectal tumors (3.2 mutations per Mb), with higher mutational loads in MSI-high tumors [48] reflecting their response to PD-1 inhibitors [49]. Low mutational loads of 1.53 mutations per Mb in RCC may reflect why PD-1 staining was not suggestive of activity to nivolumab [50]. Cytokine-based immunotherapies have also shown limited benefit in the advanced setting of sarcomas. A large randomized trial of adjuvant interferon maintenance in resected osteosarcoma patients did not provide significant improvement [51]. DSRCT is not a very immunogenic tumor. Some soft tissue and bone sarcomas have been shown to express PD-1 ligand, and additional information is emerging about the role of somatic mutations in predicting response [46, 51, 52].

There is recent evidence showing SLFN11 mRNA transcript and protein levels in DSRCT-1 are comparable to EWS cell lines. Schlafen-11 (SLFN11), a putative biomarker for defective DNA damage repair, and SCRT-1 demonstrated sensitivity to PARPi as single agent or in combination with either the topoisomerase I inhibitor irinotecan or ionizing radiation [53].

## 8. Treatment

Aggressive attempts at R0 resection have been a cornerstone of any curative intent strategy for DSRCT. HIPEC has been shown to optimize outcomes in single-center retrospective studies for disease in the abdomen [54]. Complete cytoreduction is performed prior to HIPEC using cisplatin. Because of large tumor sizes on clinical presentation and unresectable metastatic disease, surgery is usually preceded by induction of neoadjuvant HD chemotherapy, which is followed by consolidation treatment with either radiation or myeloablative treatment (Table 1) [6, 29, 55, 56].

The surgical goal is to remove >90% of the tumor [56], and resection to less than 1.0 cm tumor size [7]. This mostly requires omentectomy, peritoneal stripping, splenectomy for hilar involvement, and local resection of the diaphragmatic peritoneum [55, 56]. Microscopic negative margins are difficult to achieve because of the desmoplastic nature of the tumor; therefore consolidative 30 Gy WAP-RT [57]/IMRT [58], HIPEC [29], [^90^Y]yttrium microspheres radioembolotherapy [59], myeloablative chemotherapy (rarely used now) [55, 56, 60], or consolidative chemotherapy has been employed in most of these studies, and relapses occur early without consolidation. R0 resection and HIPEC to sterilize extensive peritoneal metastasis can lead to median survival of up to 63 months [54].

DSRCT is somewhat alkylator sensitive, and response seems to be dose responsive [55]. Doxorubicin is a common thread in the treatment of patients who either achieved long-term survival or had response to a standard consolidative radiotherapy dose of 30 Gy when delivered by external beam to the whole abdomen and pelvis [57, 58]. Myeloablative chemotherapy with thiotepa and carboplatin, etoposide followed by autologous bone marrow, or peripheral stem cell rescue has been employed with limited success [56, 61]. Case report of [^90^Y]yttrium microspheres radioembolotherapy leading to a dramatic sustained reduction in the hepatic metastatic load has also been reported [59, 62].

Most of the early case reports in the last 2 decades have used standard dose alkylating agents, Adriamycin-based treatment with less than favorable responses [3, 9, 63]. Irinotecan and temozolomide combination has shown up to 68% objective response in recurrent Ewing sarcoma during early retrospective studies [64]. Phase II study (TEMIRI) of temozolamide 100–125 mg/m^2^/day (days 1–5) and irinotecan 10 mg/m^2^/day (days 1–5 and 8–12) every 3 weeks show responses between 33% in a familiar tumor histology of medulloblastoma with some of the patients having a desmoplastic variant [65].

Kushner et al. reported 10 patients prospectively that were the first to use high-dose alkylator-based therapy (Table 1) in an alternating 7 courses of chemotherapy regimen in 1996. The P6 regimen consisted of high-dose cyclophosphamide, doxorubicin, and vincristine (HD-CAV) on cycles 1, 2, 3, and 6 given with cyclophosphamide (4200 mg/m^2^), doxorubicin (75 mg/m^2^), and vincristine (HD-CAV) alternating with ifosfamide (9 to 12 mg/m^2^) and etoposide (500 to 1000 mg/m^2^) on cycles 4, 5, and 7. The regimen was chosen due to its prior effectiveness and experience of use in Ewing sarcomas and metastatic neuroblastoma in children and young adults where it was called the “N6” protocol; N likely represents neuroblastoma [66, 67].

A modified P6 regimen and a modified PAVEP regimen [63, 68] (cyclophosphamide, pirarubicin, etoposide, and cisplatin) have been employed to decrease severe adverse events and to improve the completion rate of chemotherapy. These modified regimens use Cytoxan of 4 g/m^2^ and replace Adriamycin with pirarubicin. The modified P6 regimen use higher ifosfamide dose (12 g/m^2^ divided for five days) instead of 9 g/m^2^ in the original P6 regimen. The addition of irinotecan, topotecan, carboplatin, and cisplatin leads to few months of stable disease at best in selected patients [56, 57].

The insensitivity of the tumor to high-dose chemotherapy may implicate a stem cell hypothesis in DSRCT [69, 70]. This may reflect on the heterogeneity of the tumor and contribute to the general difficulty in eradicating the tumor. Unlike Ewing sarcoma, the putative CD133+ stem cell has not been identified to date [69, 70]. Quantitative real-time PCR analysis of putative stem cell maintenance revealed that CD133+ ESFT cells express significantly higher levels [70]. This could certainly explain tumor characteristics and lead to the identification of new targets for more effective therapies [70]. Radiation is more easily tolerated in pediatric patients and may improve local control [57, 58]. Most relapses are intraperitoneal and/or hepatic WAP-RT. Acute toxicities are approximately 80%, and almost a third of patients experience acute hematologic toxicity, with grade 4 thrombocytopenia seen in 76% of patients. Small bowel obstruction occurred in 7 patients (33%) after surgery and WAPI [57]. In one study, postoperative WAP-RT was predictive of 3-year overall survival, as were the absence of EPM and complete surgical resection. Heated intraperitoneal chemoinfusion with cisplatin had no impact on overall survival in that analysis [1].

## 9. Targeted Agents

It is unclear if, despite poor long-term outcomes, we should continue treating these patients with HD chemotherapy [2] and prolonged in-patient hospital protocols. A standard Ewing sarcoma alternating VAC/IE protocol with standard alkylator doses (Cytoxan 1200 mg/m^2^ over 60 min) and 1800 mg of ifosfamide per square meter per day for five days [71], given with mesna, could be evaluated since oncogene activation pathways in DSRCT may be similar to those in Ewing sarcoma [72]. Some centers are using a modified P6 protocol [2, 68] which is similar to VDC/IE (vincristine, total dose of 2 mg; Adriamycin, 75 mg/m^2^; and cyclophosphamide, 1200 mg/m^2^ with mesna). Dactinomycin at 1.25 mg per square meter per dose is substituted for doxorubicin when a total doxorubicin dose of 375 mg per square meter is reached. Ifosfamide and etoposide are administered (1800 mg/m^2^ of ifosfamide for five days, given with mesna, and 100 mg/m^2^ of etoposide over five days) [71]. The Ewing sarcoma regimen whether used in a dose-dense or three weekly schedule also provides a maintenance phase of treatment of up to 49 weeks [71]. There is suggestion of longer outcomes with an outpatient maintenance therapy that consisted of irinotecan and temozolomide followed by XRT and HIPEC in a 5-year-old patient [73].

Small molecule TKIs have shown dismal results so far including sorafenib and sunitinib. In a DSRCT cell line, the mTOR inhibitor induces apoptosis [74]; in practice, however, rapamycin and temsirolimus have had limited PFS [75, 76]. Therefore, mTOR inhibition may only have a role in a combination setting rather than as single therapy. In a retrospective review of patients who received pazopanib within EORTC trials, a clinical benefit rate (PR + SD >12 weeks) of 78% was reported among patients who had progressed on prior treatments among 9 patients [77].

Recently, olaratumab, a novel PDGFRa inhibitor, was approved with doxorubicin in soft tissue sarcomas (STS) with a histology subtype for which an anthracycline-containing regimen may be appropriate; however, in the study, DSRCT was not represented [78]. DSRCT had more limited representation with pazopanib approval in the PALETTE trial [79], with eribulin [80], and with the approval of trabectedin [81].

Eribulin has shown activity in pretreated patients with L-sarcomas and recently showed a 2-month survival benefit in the phase III study compared to dacarbazine [80]; however, outcomes in pretreated patients with synovial sarcoma and other types of soft tissue sarcoma did not meet the prespecified primary efficacy endpoint for activity [82]. Ewing family tumors were excluded in the study; however, three-quarters of patients were still alive at 6 months [82], suggesting that microtubule inhibition may warrant further study since vinca alkaloids have historically shown activity with Ewing family tumors [83, 84]. We must perform tumor biomarker evaluations in these clinical trials comparing responding patients with nonresponders to understand who may truly benefit or not from these therapies [83].

Gemcitabine and docetaxel have been used as an outpatient regimen for STS other than leiomyosarcoma and could have benefit in patients unable to tolerate very aggressive chemotherapy [85–87]. A clinical trial undergoing (NCT01532687) is currently looking at gemcitabine with or without pazopanib and is currently recruiting.

IGF-1R inhibition has been seen to mitigate mTOR activation and is supported by preclinical data supporting its additive antitumor effects by combining them [88]. Cixutumumab at 6 mg/kg IV weekly was combined with temsirolimus in heavily pretreated patients with Ewing family tumors that included DSRCT with one-third of the patients achieving relatively durable CR/PR [89]. This was well tolerated, with preliminary evidence of durable antitumor activity, and attempts to evaluate response in a phase II study for STS after stratifying for the expression of IGF-1R on tumor tissue [90]. Other DSRCT targeted agents include GD2 [91] and ganitumab, a fully human monoclonal antibody against type-1 insulin-like growth factor receptor (IGF-1R), showing 6% ORR and 17 (49%) SD rate in an open label phase II trial [92]. These novel clinical trials with biomarker and molecular data-driven interventions reflect the direction this field is moving with the availability of newer diagnostic tools.

## 10. Role of Immunotherapy

Tumor mutational load (TML) may affect response rates to immunotherapy as seen in NSCLC and melanoma. Higher TML tumors are more responsive to immune checkpoint inhibition [52]. Single-agent anti-PD-1 antibodies have had limited efficacy across sarcomas to date. A phase II study (SARC028) is evaluating the role of pembrolizumab across various sarcoma histologies (NCT02301039) [47]. None of the patients in a recently reported DSRCT cohort had identifiable tumoral PD-L1 expression by SP142 antibody testing, and the significance of PD-1-positive TILs is unclear at this time [43]. The composite of tumoral PD-L1 positivity and PD-1 positivity among tumor-infiltrating lymphocytes has been suggested as an indicator of prognosis in soft tissue sarcoma patients [45]. Another small, but heterogeneous, patient cohort at MSKCC demonstrated no association between PD-L1 expression, TIL and clinicopathological features, and overall survival using the DAKO 5H-1 antibody [93]. DSRCT patients, however, were not represented in these small data sets. B7H3, an immunomodulatory cell surface molecule, is seen in >90% DSRCTs. In a phase I study, a radioimmunoconjugate showed promise in an ongoing clinical trial (NCT01099644).

## 11. Future Directions

An ongoing NCT01189643 trial is looking at addition of two cycles of irinotecan, temozolomide, and bevacizumab followed by a standard P6 protocol utilizing the data, suggesting that VEGFR-2 and VEGFA overexpress in DSRCT cell lines and xenograft models [94]. A pilot study evaluating the combination of irinotecan, temozolomide, and bevacizumab is active in patients with DSRCT, and it is feasible to combine these agents with standard chemotherapy without greater than expected toxicity with response rates around 27% [95].

A phase I/II clinical trial is studying the side effects and the most effective dose of the notch signaling pathway inhibitor RO4929097 when given together with vismodegib in DSRCT patients (NCT01154452). A study looking at intraperitoneal radioimmunotherapy with a novel antibody 8H9 for patients with DSRCT is also recruiting (NCT01099644).

A current study which is ongoing but not recruiting adds irinotecan, temozolomide, and bevacizumab to the chemotherapy regimen currently used in DSRCT. An ongoing phase II study (SARC028) is looking at the role of pembrolizumab in sarcoma (NCT01189643). Similar to many general sarcoma studies, DSRCT is not represented in this study because of the limited number of patients with this disease.

## 12. Conclusion

Because of the rarity of DSRCT, limited data are available regarding the impact of various treatment modalities on survival. Aggressive surgery, radiotherapy, and chemotherapy have all been used to control DSRCT. Unfortunately, durable responses are limited, and the prognosis for patients with DSRCT remains poor [1]. The largest available single-institution study of 66 patients with DSRCT reported a 3-year and 5-year overall survival rate of 44% and 15%, respectively. Use of a combined surgery and a Ewing-based chemotherapy regimen of vincristine, doxorubicin, and cyclophosphamide (VAC) and ifosfamide + etoposide (IE) in various combinations achieves a maximal tumor debulking and is associated with improved overall survival relative to other chemotherapy regimens. Greater than 90% tumor resection was highly significant in prolonging overall survival compared to lesser resections [56]. The impact of optimal debulking was also confirmed in these studies [7, 29].

High-dose chemotherapy, radiotherapy to high-risk sites, and myeloablative chemotherapy with stem cell rescue have been described in selected cases [55]. Some investigators have described the use of cytoreduction and hyperthermic intraperitoneal chemotherapy using cisplatin for treatment of carcinomatosis and yttrium microspheres for treatment of liver metastasis from DSRCT [7]. Consolidative IMRT after debulking and/or HIPEC although used can lead to suboptimal outcomes secondary to GI and hematological toxicities and inferior DFS [96, 97]. Based upon the available data, the treatment strategy currently associated with the best overall survival includes optimal resection of ≥90% of the tumor and high-dose chemotherapy regimens. Given the significant tumor response seen in many patients following systemic chemotherapy, deferring resection until a maximal response to systemic therapy is achieved is currently advocated by some clinical investigators [62].

Little progress has been made in the field of small molecule TKIs for sarcomas since the approval of imatinib for GIST in 2002, and despite the recent FDA approval of the multi-tyrosine kinase inhibitor pazopanib, any direct efficacy for DSRCT is limited and from small retrospective studies. Rather than pursuing different chemotherapy combinations without a solid genomic basis, the field has moved to patient selection based on identifying the optimal combination of targeted therapy, chemotherapy based on chemotherapy sensitivity studies and possibly for high mutational load patient checkpoint inhibitors, or immunotherapy using a tumor signature to determine an approach so as to improve outcomes in clinically applicable ways.

A collaborative effort to include DSRCTs in clinical trials with targeted agents is crucial to determine if there truly is a clinical benefit from this novel treatment option. Recently concluded trials are eagerly awaiting to provide insight into these questions (Table 2) to show a hitherto unsurpassed survival benefit of 26.5 months in SRS with the drug olaratumab and have prompted an accelerated FDA approval in October 2016.

It is unlikely that combinational chemotherapy will significantly improve outcomes in DSRCT. Surgery should remain the cornerstone of treatment. Extended genome sequencing and immunotherapy are being assessed in future clinical trials (Table 3), and it remains to be determined what the role will be in the future for many of the emerging agents.

## Figures and Tables

**Table 1:** Summary of patients' characteristics, treatments, and outcome in DSRCT.

Study	Number of patients	Type of study	Age range	Chemo	Cytoxan dose	Response	Survival	Additional Rx	Comment
Kushner et al. [55]	10 (untreated patients)	Prospective	7–22 (median, 14 yrs)	P6	4.2 g/m^2^ over 2 days	PR 70%; CR 20% (no path CR)	Median OS 19 mo (22 for 7 pts in CR). 5 remained in CR at 38 mo	40% RT; 30% BMT; 30% ABMT^#^	1 tumor-related Budd-Chiari death. Carboplatin/thiotepa for myeloablative transplant

Hayes-Jordan et al. [29]	24	Retrospective	8–43 (median, 12 yrs)	P6	4.2 g/m^2^ over 2 days	RR not reported. Complete resection to less than 1.0 cm tumor size was achieved in all 8 patients who underwent HIPEC	3 yr OS: HIPEC + Sx = 71%; chemo/RT = 26%; Sx alone = 62%^∗∗^	HIPEC cisplatin	HIPEC only used in 5–25 yr age group. Thoracic metastasis suggested poor prognosis

Lal et al. [56] (MSKCC)	66	Retrospective	7–58 (median, 19 yrs)	P6	4.2 g/m^2^ over 2 days	Not reported	3 yr OS 44%; 5 yr OS 15%; 3 yr OS 58% with GTR^∗^	CPT-11, topotecan, carboplatin, cisplatin were added in selected patients	In 71%, greater than 90% tumor resection was possible. 71% underwent Rx with P6 regimen

Farhat et al. [63]	5	Retrospective	16–26 (median 22 yrs)	PA(E)VP	900 mg/m^2^ over 3 days	4 stable disease 1 CR	Mean survival 24 mo	ABMT (carboplatin, 800 mg/m^2^; etoposide, 1200 mg/m^2^; and ifosfamide, 6 g/m^2^) in 1 patient	Chemotherapy was given adjuvantly. 1 CR was reported to have tunica vaginalis (primary)

Pinnix et al. [58]	8	Retrospective	5–20 (median, 11 yrs)	P6	4.2 g/m^2^ over 2 days	5/8 had complete resection; 2/8 had near complete (>90%) resection	At 30 mo, three patients died of PD, four were alive with active disease, and one was in CR	7/8 patients had HIPEC	25% had extra-abdominal metastasis. Mean time to IMRT failure 6.6 mo. 70–80% Gr 2 GI toxicity. Limited Gr ½ hematological toxicity mostly anemia

Goodman et al. [57] (MSKCC)	21	Retrospective	8–34 (median, 16.5 yrs)	P6	4.2 g/m^2^ over 2 days	Not reported. Maximal debulking in all but 1 patient	3 yr OS 48%; 3 yr RFS 14%; median OS 32 mo	Cisplatin, carboplatin, topotecan, irinotecan, and vinorelbine were also used. 30 Gy WA-XRT	Grade 4 thrombocytopenia, leukopenia, and anemia in 76%, 29%, and 33%, respectively. Bowel obstruction in 33%

Wong et al. [98]	41	Retrospective	16–45 (median, 27 yrs)	Vincristine + ifosfamide + doxorubicin + etoposide (VIDE) in a 1/3rd of 1st line Rx	Ifos 3 g/m^2^ over 3 days	Not reported	3 yr OS 27%; 5 yr OS 16%	6/41 received XRT	VIDE chemotherapy appeared to confer the longest TTP (median, 14.6 months)

Aguilera et al. [73]	1 (5 yr old, only outpatient regimen)	Case report	5 yrs	VIDE (vincristine (1.5 mg/m^2^), dexrazoxane/doxorubicin (750/75 mg/m^2^), and etoposide (150 mg/m^2^))	Ifos 3 g/m^2^ over 3 days (outpatient)	R0 resection with microscopic residual disease	Relapse at 18 months. Alive at 2 yrs after Dx	HIPEC cisplatin 100 mg/m^2^ and aggressive tumor debulking. Followed by Temodar/irinotecan maintenance x12 followed by IMRT (30 Gy)	Ifosfamide infusions were done at home with bag changes by home health nursing. Retroperitoneal relapse treated with IMRT with bevacizumab (5 mg/kg) and 2 perihepatic metastases with radiofrequency ablation/cryoablation followed by chronic outpatient maintenance chemotherapy (valproic acid, cyclophosphamide, and rapamycin)

^#^ABMT = autologous myeloablative transplant. ^∗^GTR = gross tumor resection. ^∗∗^There was no statistical difference in estimated OS for those who received debulking surgery compared with HIPEC, in those who did not receive HIPEC. There were no survivors greater than 3 years.

**Table 2:** Clinical trials recently completed in DSRCT.

Clinical trial (ID); phase	Drugs	Status	Assigned intervention
NCT01154452; phase 1B/II	Vismodegib (hedgehog inhibitor) and NOTCH inhibitor RO4929097	Completed	Vismodegib and Gamma-Secretase/Notch Signalling Pathway Inhibitor RO4929097 in Treating Patients with Advanced or Metastatic Sarcoma

NCT00563680; phase II	Drug: AMG 479 (IGF-R1 Ab)	Completed	QUILT-3.025: A Phase 2 Study of AMG 479 in Relapsed or Refractory Ewing's Family Tumor and Desmoplastic Small Round Cell Tumors

NCT00062205; phase I, II	Drug: imatinib mesylate	Completed	Imatinib Mesylate in Treating Patients With Recurrent Ewing's Family of Tumors or Desmoplastic Small Round Cell Tumor

NCT00055952; phase II	Drug: exatecan mesylate (camptothecin)	Completed	Exatecan Mesylate in Treating Patients With Ewing's Sarcoma, Primitive Neuroectodermal Tumor, or Desmoplastic Small Round Cell Tumor

NCT00720174; phase I	Biological: cixutumumab (IGF-1R Ab); drug: doxorubicin hydrochloride; other: laboratory biomarker analysis	Completed	Cixutumumab and Doxorubicin Hydrochloride in Treating Patients With Unresectable, Locally Advanced, or Metastatic Soft Tissue Sarcoma

NCT00436657; phase I	Drug: CHPP of cisplatin; procedure: abdominal surgery	Completed	Continuous Hyperthermic Peritoneal Perfusion (CHPP) With Cisplatin for Children With Peritoneal Cancer

NCT00093821; phase I	Drug: tanespimycin (HSP90 inhibitor)	Completed	Tanespimycin in Treating Young Patients With Recurrent or Refractory Leukemia or Solid Tumors

**Table 3:** Ongoing clinical trials in DSRCT.

Clinical trial (ID); phase	Drugs	Current status	Assigned intervention
NCT01189643; pilot study	CPT-11, TMZ, bevacizumab	Ongoing but not recruiting	Two cycles of the investigational combination irinotecan, temozolomide, and bevacizumab will be given followed by conventional chemotherapy with a modified P6 approach and surgical local control. Completion of modified P6 chemotherapy will be followed by a second-look surgery

NCT01099644; phase I	Biological: ^131^I-8H9	Recruiting	Intraperitoneal Radioimmunotherapy With ^131^I-8H9 for Patients With Desmoplastic Small Round Cell Tumors and Other Solid Tumors Involving the Peritoneum

NCT02173093; phase I	Biological: IL-2 | biological: GD2Bi-aATC | biological: GM-CSF	Recruiting	Activated T Cells Armed With GD2 Bispecific Antibody in Children and Young Adults With Neuroblastoma and Osteosarcoma, DSRCT

NCT02982941; phase I	Drug: enoblituzumab	Recruiting	Enoblituzumab (MGA271) in Children With B7-H3-expressing Solid Tumors

NCT01532687; phase II	Gemcitabine ± pazopanib	Recruiting	Gemcitabine Hydrochloride With or Without Pazopanib Hydrochloride in Treating Patients With Refractory Soft Tissue Sarcoma

NCT00089245; phase I	Radiation: iodine I-131 monoclonal antibody 8H9	Recruiting	Radiolabeled Monoclonal Antibody Therapy in Treating Patients with Refractory, Recurrent, or Advanced CNS or Leptomeningeal Cancer/Sarcomas
